# Catheter Ablation in Patients with Electrical Storm in Early Post Infarction Period (6 Weeks): A Single Centre Experience

**DOI:** 10.1016/s0972-6292(16)30794-x

**Published:** 2014-10-06

**Authors:** Daljeet Saggu, Mandar Shah, Arun Gopi, Abdhija Hanumandla, Calambur Narasimhan

**Affiliations:** CARE Hospital, The Institute of Medical Sciences, Hyderabad, India

**Keywords:** Electrical storm, Myocardial Infarction, Catheter Ablation

## Abstract

**Background:**

Electrical storm (ES) due to drug refractory ventricular tachycardia (VT) occurring within first few weeks of acute myocardial infarction (MI) has poor prognosis. Catheter ablation has been proposed for treating VT occurring late after MI, but there is limited data on catheter ablation in VT within first few weeks of MI.

**Methods and Results:**

Five patients (4 males, mean age 54.2±12.11 years) between June 2008 to July 2012, referred for VT presenting as ES refractory to antiarrhythmic drugs in the early post infarction period (six weeks following MI) despite revascularization. Three patients had anterior wall MI and two inferior wall MI with left ventricular ejection fraction ranging from 26 to 35%.All underwent catheter ablation within 48 hours of being in VT except one who presented late. Clinical VT was induced in all five patients. Total number of VTs induced were 11 (2.2±1.09 per patient). Two patients needed epicardial ablation via pericardial puncture. Though acute success was 100%, one patient had recurrence of clinical VT the next day of procedure.One patient succumbed to sepsis with multiple organ failure. The remaining four patients are doing well without further clinical recurrence of VT over a period of 3.7 years of follow-up.

**Conclusion:**

Catheter ablation can be a useful adjunctive therapy for patients with recurrent VT in the early post infarction period. This procedure appears to be safe with acceptable success rate.

## Introduction

The occurrence of malignant ventricular arrhythmias during the subacute phase of MI in patients with ventricular dysfunction has a poor outcome. [1] Although there are several reports of catheter ablation of VT late after MI, there is little information regarding VT ablation in the early phase of MI. We report five patients who underwent successful catheter ablation of recurrent drug refractory VT in the early post infarction period which was defined as VT occurring within 6 weeks of MI.

## Methods

From June 2008 to July 2012, five patients were referred for VT presenting as ES in the early post infarction period despite revascularization. All the five patients were refractory to multiple antiarrhythmic drugs (amiodarone, lidocaine, beta blocker and magnesium) and had received multiple cardioversions for termination of VT. Metabolic, acid-base and electrolyte abnormalities were corrected. One patient who already had an implantable cardioverter defibrillator (ICD) was referred to us for electrical storm for more than 48 hours, had developed pre-renal azotemia and was on maintenance hemodialysis for the same. All the five patients underwent emergent catheter ablation under 3D electroanatomic mapping (EAM) within 48 hours of presentation except one.

### Mapping and ablation

**Figure 1:**
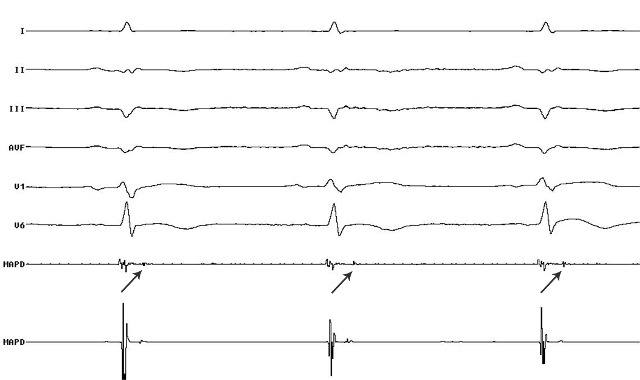
Patient 2; surface ECG and Intracardiac: MAPD - arrows showing isolated late potentials during substrate mapping

**Figure 2:**
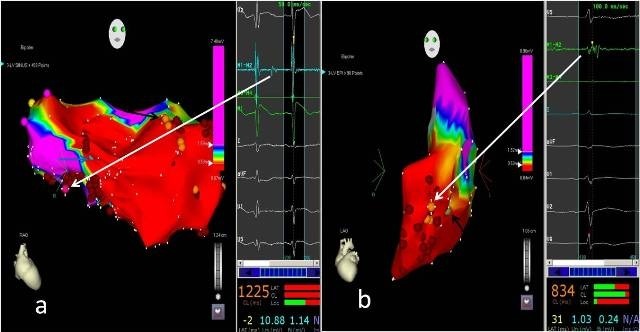
a, Endocardial electroanatomic bipolar voltage map of LV of patient number 4 in RAO projection. Dark pink tag represents isolated late potential (white arrow), orange tags (black arrow) represents fractionated signals and blue tag (blue arrow) represents double potentials at which sites ablation was done (red tags). Pink tag represents annulus and yellow tag represents His Bundle. b, Epicardial electroanatomic bipolar voltage map of LV of patient number 1 in LAO projection. The orange tags represent fractionated signals (white arrow) and green tag (black arrow) represent good pace map at which sites ablation was done (red tags). LV- left ventricle; RAO- right anterior oblique; LAO- left anterior oblique.

**Figure 3:**
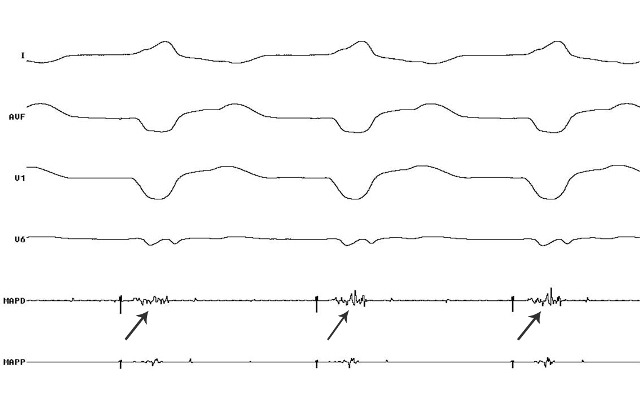
Patient 3; surface ECG and Intracardiac - MAPD showing fractionated potential during V pacing suggestive of site of slow conduction

**Figure 4:**
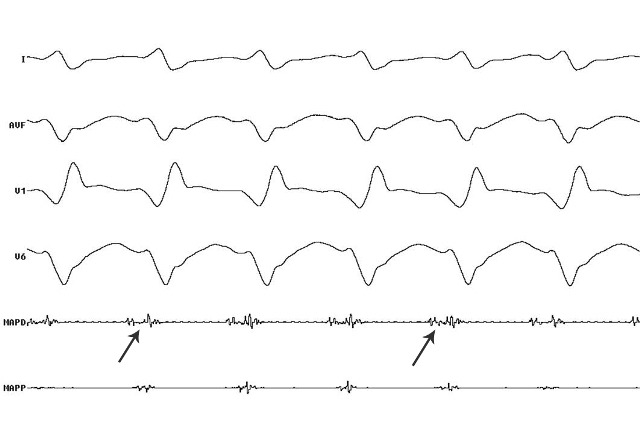
Patient 2; surface ECG and Intracardiac signals; MAPD - arrows showing pre-systolic potential during VT, site of successful ablation

The procedure was done under local anesthesia and conscious sedation. The patient with an ICD, was taken up after the tachycardia therapy was switched off. Vascular access was obtained via right femoral vein and artery. Quadripolar catheters were positioned in the region of His bundle and right ventricle / right atrium through femoral venous access. CARTO 3D EAM system with a 3.5 mm open irrigated tip catheter (Navistar, Biosense Webster) was used for mapping and ablation. Left ventricular access was obtained by using a retrograde aortic approach in all 5 patients. During substrate mapping, areas of low voltage, isolated late potentials (Figure 1 and Figure 2a) and fractionated potentials (Figure 2b and 3) were tagged and ablated in the scar area and peri-scar area. Unstable VTs were mapped during sinus rhythm with pace mapping. Bipolar pace-mapping was performed at 10 mA and a pulse width of 2 ms. During activation mapping, mid-diastolic or pre-systolic potentials were sought, entrainment was performed and critical isthmus of the VT re-entrant circuit defined. radiofrequency (RF) energy was delivered at sites where there was concealed entrainment with stimulus to QRS nearly equal to electrogram to QRS duration and post pacing interval nearly identical to tachycardia cycle length (TCL). If the tachycardia could not be entrained, the earliest local ventricular electrograms were targeted (Figure 4). The site where tachycardia terminated was labelled as site of successful ablation.

RF energy was delivered at a power of 30-40 W with a temperature limit of 45-55ºC targeting an impedance drop of 10 Ohms. If delivery of RF energy during tachycardia failed to terminate the VT within 30 seconds, the catheter was moved to an alternate site. If the tachycardia terminated during energy delivery, the energy was continued for 60 seconds and energy was delivered at the same site (60 to 90 seconds each) for consolidating the lesion. Drop of impedance by 10 Ohms, decrease in amplitude of the electrogram signal and inability to capture the myocardium at the site where there was reliable capture pre-ablation served as indicators for adequate energy delivery at the said site. After ablation, the entire stimulation protocol was repeated at two right ventricular sites. If VTs remained inducible, further mapping and ablation were performed until a critical site was no longer identifiable.

## Results

The patients included in the study were aged 54.2±12.11 years. Four patients were male. Baseline characteristics are given in table 1. All the five patients had significant left ventricular systolic dysfunction, with left ventricular ejection fraction ranging from 26 to 35%. These patients had recurrent VT in the early post-infarction period despite multiple antiarrhythmic drugs and other medical management. VT occurred (beyond first 48 hours) 17, 5, 15, 33 and 40 days (22±14.2 days) after the infarction. Three patients had a single clinical VT morphology while two patients had two different morphologies (Table 1). At least one of the tachycardia episodes in each patient was hemodynamically unstable, requiring electrical cardioversion. All patients were taken up for ablation within 24 hours of presentation to our institute.

Clinical VT was induced in all five patients. Additionally, one or more morphologies of non-clinical VT were induced in four patients (Table 2). The total number of VTs induced was 11 (2.2±1.09 per patient). Substrate mapping was performed in all the five patients. Additionally, activation mapping was performed in three patients. Along with endocardial mapping and ablation, two patients needed epicardial ablation which was done via percutaneous pericardial puncture. Two patients who underwent activation mapping had Purkinje potentials earlier than the surface QRS and earlier than the local V at this site during VT suggesting participation of Purkinje fibers. Ablation at these sites during sinus rhythm eliminated the potentials. After this, no tachycardia could be induced.

Acute success, as defined by non-inducibility of any monomorphic VT, either clinical or non-clinical was 100% but patient 4 had recurrence of clinical VT the next day of procedure which was hemodynamically stable and responded to intravenous amiodarone. There were no complications related to the procedure. Patient number 1 who was referred to us after 48 hours of being in electrical storm with multiple ICD shocks with prerenal azotemia although underwent successful ablation of VT without further recurrences, developed multiple organ failure and eventually succumbed to the same during the course of hospital stay. Out of the remaining four patients ICD implantation was done in only one because of financial constraints. All these four patients have completed mean of 45.5 months of follow-up without any further recurrences of VT.

## Discussion

Electrical storm due to recurrent VT presenting after 24 hours but within few weeks of acute MI is rare but difficult to treat. [2] Overall mortality with antiarrhythmic drugs is more than 50%. [2] The use of sympathetic blockade with left stellate ganglionic block and beta blockade can decrease the mortality by up to 22%. [1] Also Bourke et al [3] have documented that initiation of thoracic epidural anaesthesia (TEA) in refractory VT decreases the arrhythmia burden by 75%. We did not use this approach in any of our patients as all of them were on dual antiplatelet drugs with recent percutaneous coronary intervention. There are a large number of clinical trials on catheter ablation of VT late after MI but data regarding safety and efficacy of RF ablation in the early post infarction period is limited.

Thoppil et al [4] reported a patient with drug refractory persistent VT two weeks after acute MI, who underwent successful catheter ablation of VT without further recurrence at 3 months follow-up. Dietmar et al [5] reported 4 patients with post MI drug refractory incessant VT being triggered by ventricular premature beats (VPBs). Successful RF ablation was done in all 4 patients targeting the triggering VPBs. No recurrence occurred for 33, 14, 6 and 5 months follow-up in patients 1, 2, 3 and 4 respectively. Older studies conducted on experimental models of acute MI have suggested different mechanisms responsible for occurrence of arrhythmias which include enhanced automaticity of subendocardial Purkinje fibers that survived MI, triggered activity arising from delayed after depolarizations and re-entry. [6-8] Two of our patients required epicardial mapping and ablation. The possible explanation for which may be that surviving strands of viable myocardial tissue may be present in the epicardial surface which serves as protected isthmus for the re-entrant VT. This is likely to occur when the wave front of ischemic area stops short of the epicardial layer. It is also possible that VT circuit is three dimensional, the entrance to the isthmus may be epicardial and exit may be endocardial. To the best of our knowledge this is the first case series of five patients with refractory VT in the early post infarction period that underwent successful catheter ablation without any procedure related complication. Although one patient has succumbed to non arrhythmic death, the other four are free of VT recurrence with an average of 3.7 years of follow-up.

## Limitation

This is a single centre experience of a small cohort of patients. Large controlled studies need to be done.

## Figures and Tables

**Table 1:**
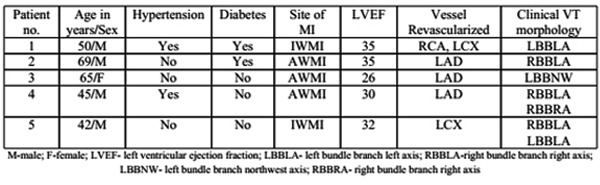
Baseline characteristics

**Table 2:**
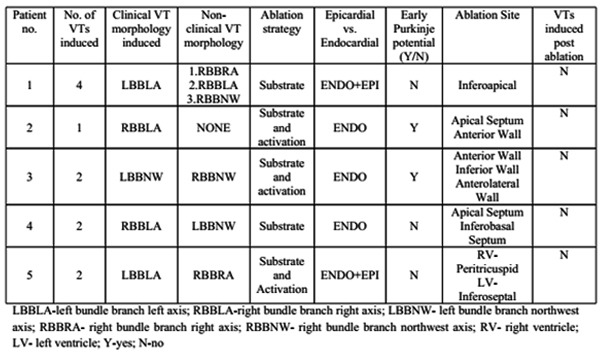
Details of the ablation procedure
